# *N*-Benzyl-4-hy­droxy-2-methyl-1,1-dioxo-2*H*-1λ^6^,2-benzothia­zine-3-carboxamide

**DOI:** 10.1107/S1600536812022805

**Published:** 2012-05-31

**Authors:** Farhana Aman, Waseeq Ahmad Siddiqui, Adnan Ashraf, Hamid Latif Siddiqui, Masood Parvez

**Affiliations:** a Department of Chemistry, University of Sargodha, Sargodha 40100, Pakistan; b Institute of Chemistry, University of the Punjab, Lahore-54590, Pakistan; c Department of Chemistry, The University of Calgary, 2500 University Drive NW, Calgary, Alberta, Canada T2N 1N4

## Abstract

In the title mol­ecule, C_17_H_16_N_2_O_4_S, the heterocyclic thia­zine ring adopts a half-chair conformation, with the S and N atoms displaced by 0.546 (4) and 0.281 (4) Å, respectively, on opposite sides of the mean plane formed by the remaining ring atoms. The mol­ecular structure is stabilized by an intra­molecular O—H⋯O hydrogen bond. The two aromatic rings are inclined to one another by 42.32 (11)°. In the crystal, mol­ecules are linked by pairs of N—H⋯O hydrogen bonds, forming inversion dimers. The dimers are linked *via* a series of C—H⋯O inter­actions, leading to the formation of a three-dimensional network.

## Related literature

For the biological activity of benzothia­zine derivatives, see: Lomabardino & Wiseman *et al.* (1972 ▶); Lazzeri *et al.* (2001 ▶). For the synthetic procedure, see: Siddiqui *et al.* (2008 ▶). For the structures of similar compounds, see: Siddiqui *et al.* (2008 ▶, 2009 ▶).
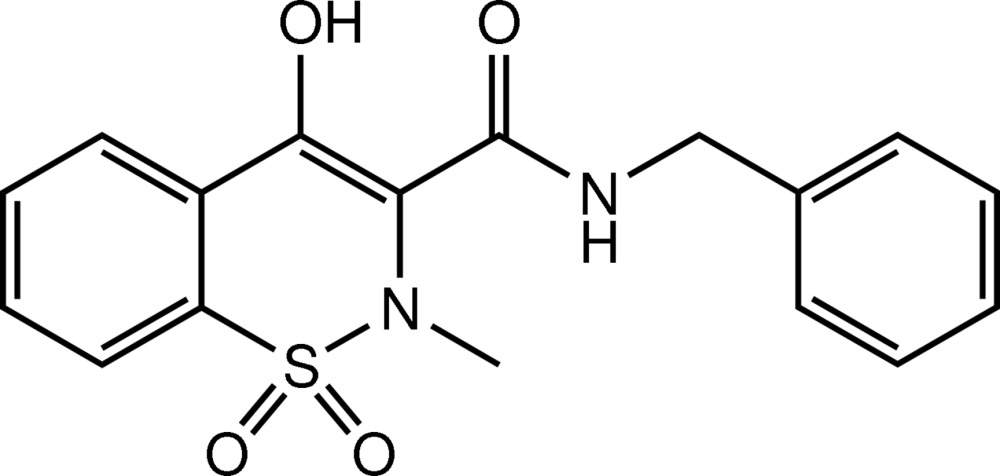


## Experimental

### 

#### Crystal data

C_17_H_16_N_2_O_4_S
*M* *_r_* = 344.38
Triclinic, 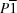

*a* = 8.785 (3) Å
*b* = 9.122 (3) Å
*c* = 11.425 (4) Å
α = 66.61 (2)°
β = 87.66 (2)°
γ = 69.38 (2)°
*V* = 781.2 (5) Å^3^
*Z* = 2
Mo *K*α radiation
μ = 0.23 mm^−1^
*T* = 173 K
0.16 × 0.16 × 0.10 mm

#### Data collection

Nonius KappaCCD diffractometer
Absorption correction: multi-scan (*SORTAV*; Blessing, 1997 ▶) *T*_min_ = 0.964, *T*_max_ = 0.977
6837 measured reflections
3592 independent reflections
3141 reflections with *I* > 2σ(*I*)
*R*_int_ = 0.028

#### Refinement

*R*[*F* ^2^ > 2σ(*F* ^2^)] = 0.046
*wR*(*F* ^2^) = 0.113
*S* = 1.06
3592 reflections
219 parameters
H-atom parameters constrained
Δρ_max_ = 0.35 e Å^−3^
Δρ_min_ = −0.42 e Å^−3^

### 

Data collection: *COLLECT* (Hooft, 1998 ▶); cell refinement: *DENZO* (Otwinowski & Minor, 1997 ▶); data reduction: *SCALEPACK* (Otwinowski & Minor, 1997 ▶); program(s) used to solve structure: *SHELXS97* (Sheldrick, 2008 ▶); program(s) used to refine structure: *SHELXL97* (Sheldrick, 2008 ▶); molecular graphics: *ORTEP-3 for Windows* (Farrugia, 1997 ▶); software used to prepare material for publication: *SHELXL97* .

## Supplementary Material

Crystal structure: contains datablock(s) global, I. DOI: 10.1107/S1600536812022805/su2428sup1.cif

Structure factors: contains datablock(s) I. DOI: 10.1107/S1600536812022805/su2428Isup2.hkl

Supplementary material file. DOI: 10.1107/S1600536812022805/su2428Isup3.cml

Additional supplementary materials:  crystallographic information; 3D view; checkCIF report

## Figures and Tables

**Table 1:** Hydrogen-bond geometry (Å, °)

*D*—H⋯*A*	*D*—H	H⋯*A*	*D*⋯*A*	*D*—H⋯*A*
O3—H3*O*⋯O4	0.84	1.79	2.531 (2)	146
N2—H2*N*⋯O1^i^	0.88	2.24	2.980 (2)	141
C10—H10*A*⋯O4^ii^	0.98	2.50	3.349 (3)	144
C11—H11*B*⋯O1^iii^	0.99	2.51	3.374 (3)	146
C15—H15⋯O2^iv^	0.95	2.59	3.496 (3)	158

Symmetry codes: (i) 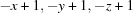; (ii) 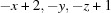; (iii) 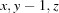; (iv) 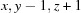.

**Fig. 1.**
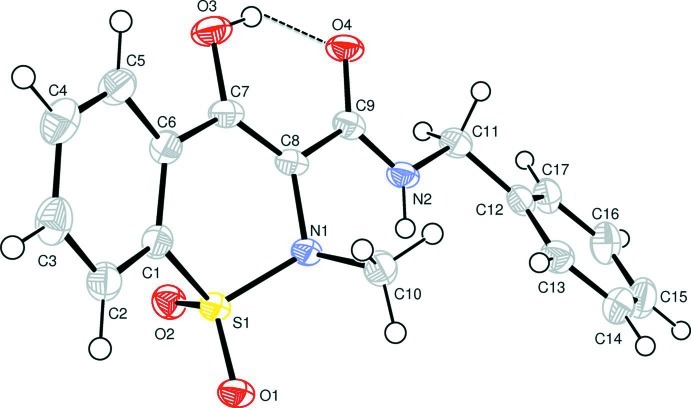
The molecular structure of the title molecule with the atom numbering. Displacement ellipsoids are drawn at the 50% probability level. The intramolecular O-H···O hydrogen bond is shown as a dashed line.

**Fig. 2.**
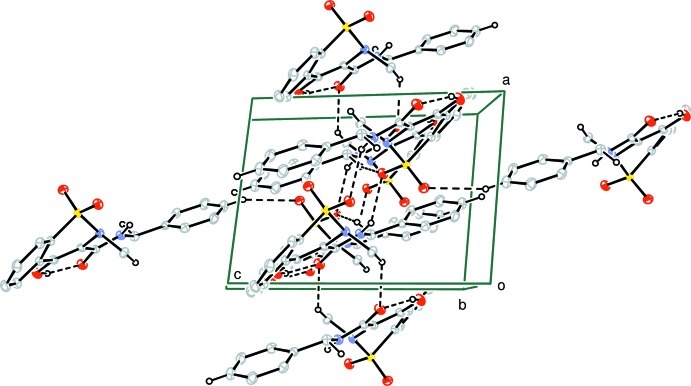
A view along the b axis of the crystal packing in the title compound, with the O-H···O, N—H···O and C—H···O hydrogen bonds shown as dashed lines [H atoms non-participating in hydrogen-bonding have been omitted for clarity].

## supplementary crystallographic information

### Comment

1,2-Benzothiazine 1,1 dioxides have received much attention since the discovery of 4-hydroxy-2-methyl-2*H*-1,2-benzothiazine-3-carboxamides 1,1-dioxides as potent anti-inflammatory and analgesic agents (Lomabardino & Wiseman, 1972). Benzothiazine based derivatives have also shown activities for the treatment of asthmatic therapy (Lazzeri *et al.*, 2001). In continuation of our interest in the synthesis and crystal structures of 1,2-benzothiazine derivatives (Siddiqui *et al.*, 2008; 2009) we report herein on the crystal structure of the title compound.

The bond distances and angles in the title compound (Fig. 1) agree very well with the corresponding bond distances and angles reported for closely related compounds (Siddiqui *et al.*, 2008; 2009). The heterocyclic thiazine ring adopts a half chair conformation with atoms S1 and N1 displaced by 0.546 (4) and 0.281 (4) Å, respectively, on the opposite sides from the mean plane formed by the remaining ring atoms. The molecular structure is stabilized by an intramolecular O3—H3O···O4 hydrogen bond (Table 1). The aromatic rings [C1-C6 and C12-C17] are inclined to one another by 42.32 (11)°.

In the crystal, molecules are linked by a pair of N—H···O hydrogen bonds to form inversion dimers. The dimers are linked via a series of C—H···O interactions leading to the formation of a three-dimensional network. (Fig. 2 and Table 1).

### Experimental

For the synthesis of the title compound, 4-hydroxy-2-methyl-2*H*- 1,2-benzothiazine- 3-carboxylic acid methyl ester 1,1 dioxide and benzyllamine were used as the starting materials following a procedure reported earlier (Siddiqui *et al.*, 2008). Crystals of the title compound, suitable for the X-ray crystallographic study, were grown from a mixture of ethyl acetate and methanol (1:1) at room temperature [M.p. = 466 – 467 K].

### Refinement

All H atoms were positioned geometrically and refined using a riding model, with O—H = 0.84 Å, N—H = 0.88 Å and C—H = 0.95, 0.98 and 0.99 Å, for aryl, methyl and methylene H-atoms, respectively, with *U*_iso_(H) = k × U_eq_(O,N,C), where k = 1.5 for OH and CH_3_ H atoms and = 1.2 for other H atoms.

### Crystal data

**Table:**

C_17_H_16_N_2_O_4_S	*Z* = 2
*M**_r_* = 344.38	*F*(000) = 360
Triclinic, *P*1	*D*_x_ = 1.464 Mg m^−^^3^
Hall symbol: -P 1	Mo *K*α radiation, λ = 0.71073 Å
*a* = 8.785 (3) Å	Cell parameters from 3525 reflections
*b* = 9.122 (3) Å	θ = 1.0–27.5°
*c* = 11.425 (4) Å	µ = 0.23 mm^−^^1^
α = 66.61 (2)°	*T* = 173 K
β = 87.66 (2)°	Prism, colourless
γ = 69.38 (2)°	0.16 × 0.16 × 0.10 mm
*V* = 781.2 (5) Å^3^	

### Data collection

**Table:**

Nonius KappaCCD diffractometer	3592 independent reflections
Radiation source: fine-focus sealed tube	3141 reflections with *I* > 2σ(*I*)
Graphite monochromator	*R*_int_ = 0.028
ω and φ scans	θ_max_ = 27.6°, θ_min_ = 3.3°
Absorption correction: multi-scan (*SORTAV*; Blessing, 1997)	*h* = −10→11
*T*_min_ = 0.964, *T*_max_ = 0.977	*k* = −11→11
6837 measured reflections	*l* = −14→14

### Refinement

**Table:**

Refinement on *F*^2^	Primary atom site location: structure-invariant direct methods
Least-squares matrix: full	Secondary atom site location: difference Fourier map
*R*[*F*^2^ > 2σ(*F*^2^)] = 0.046	Hydrogen site location: inferred from neighbouring sites
*wR*(*F*^2^) = 0.113	H-atom parameters constrained
*S* = 1.06	*w* = 1/[σ^2^(*F*_o_^2^) + (0.0393*P*)^2^ + 0.7103*P*] where *P* = (*F*_o_^2^ + 2*F*_c_^2^)/3
3592 reflections	(Δ/σ)_max_ < 0.001
219 parameters	Δρ_max_ = 0.35 e Å^−^^3^
0 restraints	Δρ_min_ = −0.42 e Å^−^^3^

### Special details

**Table:**

Geometry. All e.s.d.'s (except the e.s.d. in the dihedral angle between two l.s. planes) are estimated using the full covariance matrix. The cell e.s.d.'s are taken into account individually in the estimation of e.s.d.'s in distances, angles and torsion angles; correlations between e.s.d.'s in cell parameters are only used when they are defined by crystal symmetry. An approximate (isotropic) treatment of cell e.s.d.'s is used for estimating e.s.d.'s involving l.s. planes.
Refinement. Refinement of *F*^2^ against ALL reflections. The weighted *R*-factor *wR* and goodness of fit *S* are based on *F*^2^, conventional *R*-factors *R* are based on *F*, with *F* set to zero for negative *F*^2^. The threshold expression of *F*^2^ > σ(*F*^2^) is used only for calculating *R*-factors(gt) *etc*. and is not relevant to the choice of reflections for refinement. *R*-factors based on *F*^2^ are statistically about twice as large as those based on *F*, and *R*- factors based on ALL data will be even larger.

### Fractional atomic coordinates and isotropic or equivalent isotropic displacement parameters (Å^2^)

**Table:**

	*x*	*y*	*z*	*U*_iso_*/*U*_eq_	
S1	0.62867 (5)	0.60505 (6)	0.34904 (4)	0.02296 (13)	
O1	0.58802 (17)	0.69700 (17)	0.42959 (14)	0.0283 (3)	
O2	0.50059 (16)	0.61698 (18)	0.26852 (14)	0.0293 (3)	
O3	0.93624 (19)	0.2725 (2)	0.19251 (15)	0.0345 (3)	
H3O	0.9365	0.1725	0.2308	0.052*	
O4	0.88028 (17)	0.02644 (18)	0.36729 (14)	0.0309 (3)	
N1	0.72650 (19)	0.40160 (19)	0.44128 (15)	0.0219 (3)	
N2	0.71929 (19)	0.0748 (2)	0.51809 (16)	0.0253 (3)	
H2N	0.6669	0.1466	0.5526	0.030*	
C1	0.7810 (2)	0.6574 (2)	0.25555 (18)	0.0249 (4)	
C2	0.8007 (3)	0.8122 (3)	0.22507 (19)	0.0296 (4)	
H2	0.7324	0.8939	0.2550	0.036*	
C3	0.9231 (3)	0.8447 (3)	0.1495 (2)	0.0357 (5)	
H3	0.9389	0.9499	0.1275	0.043*	
C4	1.0220 (3)	0.7253 (3)	0.1060 (2)	0.0375 (5)	
H4	1.1040	0.7501	0.0534	0.045*	
C5	1.0029 (3)	0.5700 (3)	0.1383 (2)	0.0323 (5)	
H5	1.0714	0.4889	0.1079	0.039*	
C6	0.8826 (2)	0.5329 (3)	0.21550 (18)	0.0262 (4)	
C7	0.8691 (2)	0.3635 (3)	0.26145 (19)	0.0257 (4)	
C8	0.7992 (2)	0.3010 (2)	0.36928 (19)	0.0235 (4)	
C9	0.8009 (2)	0.1249 (2)	0.41831 (19)	0.0248 (4)	
C10	0.8312 (3)	0.3639 (3)	0.55606 (19)	0.0291 (4)	
H10A	0.8803	0.2394	0.6056	0.035*	
H10B	0.7645	0.4172	0.6096	0.035*	
H10C	0.9182	0.4100	0.5288	0.035*	
C11	0.7160 (3)	−0.0979 (2)	0.5706 (2)	0.0287 (4)	
H11A	0.8282	−0.1813	0.5792	0.034*	
H11B	0.6461	−0.1068	0.5098	0.034*	
C12	0.6520 (2)	−0.1446 (2)	0.69982 (19)	0.0272 (4)	
C13	0.6713 (3)	−0.0783 (3)	0.7863 (2)	0.0317 (4)	
H13	0.7232	0.0031	0.7634	0.038*	
C14	0.6155 (3)	−0.1299 (3)	0.9056 (2)	0.0392 (5)	
H14	0.6304	−0.0845	0.9641	0.047*	
C15	0.5382 (3)	−0.2469 (3)	0.9401 (2)	0.0437 (6)	
H15	0.4990	−0.2810	1.0216	0.052*	
C16	0.5185 (3)	−0.3139 (3)	0.8547 (2)	0.0411 (5)	
H16	0.4660	−0.3948	0.8779	0.049*	
C17	0.5749 (3)	−0.2635 (3)	0.7358 (2)	0.0322 (5)	
H17	0.5610	−0.3104	0.6780	0.039*	

### Atomic displacement parameters (Å^2^)

**Table:**

	*U*^11^	*U*^22^	*U*^33^	*U*^12^	*U*^13^	*U*^23^
S1	0.0241 (2)	0.0199 (2)	0.0242 (2)	−0.00610 (17)	0.00430 (17)	−0.01015 (18)
O1	0.0335 (7)	0.0217 (7)	0.0312 (7)	−0.0075 (6)	0.0082 (6)	−0.0148 (6)
O2	0.0265 (7)	0.0291 (7)	0.0301 (7)	−0.0081 (6)	0.0004 (6)	−0.0116 (6)
O3	0.0401 (8)	0.0339 (8)	0.0351 (8)	−0.0121 (7)	0.0151 (7)	−0.0218 (7)
O4	0.0336 (7)	0.0250 (7)	0.0369 (8)	−0.0068 (6)	0.0096 (6)	−0.0192 (6)
N1	0.0251 (8)	0.0179 (7)	0.0232 (8)	−0.0065 (6)	0.0040 (6)	−0.0101 (6)
N2	0.0270 (8)	0.0200 (8)	0.0295 (9)	−0.0057 (6)	0.0052 (7)	−0.0134 (7)
C1	0.0275 (9)	0.0249 (9)	0.0208 (9)	−0.0096 (7)	0.0025 (7)	−0.0081 (7)
C2	0.0369 (11)	0.0266 (10)	0.0253 (10)	−0.0127 (8)	0.0026 (8)	−0.0096 (8)
C3	0.0484 (13)	0.0329 (11)	0.0288 (11)	−0.0236 (10)	0.0048 (9)	−0.0079 (9)
C4	0.0395 (12)	0.0447 (13)	0.0296 (11)	−0.0222 (10)	0.0101 (9)	−0.0112 (10)
C5	0.0322 (10)	0.0371 (11)	0.0269 (10)	−0.0121 (9)	0.0079 (8)	−0.0132 (9)
C6	0.0264 (9)	0.0275 (10)	0.0229 (9)	−0.0089 (8)	0.0032 (7)	−0.0093 (8)
C7	0.0228 (9)	0.0275 (10)	0.0284 (10)	−0.0058 (7)	0.0040 (7)	−0.0160 (8)
C8	0.0211 (8)	0.0222 (9)	0.0277 (9)	−0.0047 (7)	0.0037 (7)	−0.0136 (8)
C9	0.0228 (9)	0.0228 (9)	0.0291 (10)	−0.0054 (7)	0.0005 (7)	−0.0132 (8)
C10	0.0342 (10)	0.0256 (10)	0.0268 (10)	−0.0085 (8)	−0.0015 (8)	−0.0115 (8)
C11	0.0334 (10)	0.0208 (9)	0.0329 (10)	−0.0088 (8)	0.0039 (8)	−0.0131 (8)
C12	0.0263 (9)	0.0210 (9)	0.0279 (10)	−0.0036 (7)	−0.0025 (8)	−0.0075 (8)
C13	0.0317 (10)	0.0290 (10)	0.0344 (11)	−0.0085 (8)	0.0005 (8)	−0.0150 (9)
C14	0.0449 (13)	0.0403 (13)	0.0286 (11)	−0.0094 (10)	−0.0024 (9)	−0.0150 (10)
C15	0.0461 (13)	0.0460 (14)	0.0266 (11)	−0.0137 (11)	0.0029 (10)	−0.0053 (10)
C16	0.0466 (13)	0.0376 (12)	0.0328 (12)	−0.0200 (11)	−0.0012 (10)	−0.0037 (10)
C17	0.0370 (11)	0.0266 (10)	0.0282 (10)	−0.0102 (9)	−0.0035 (8)	−0.0072 (8)

### Geometric parameters (Å, º)

**Table:**

S1—O2	1.4293 (15)	C5—H5	0.9500
S1—O1	1.4328 (14)	C6—C7	1.468 (3)
S1—N1	1.6404 (17)	C7—C8	1.359 (3)
S1—C1	1.757 (2)	C8—C9	1.471 (3)
O3—C7	1.340 (2)	C10—H10A	0.9800
O3—H3O	0.8400	C10—H10B	0.9800
O4—C9	1.255 (2)	C10—H10C	0.9800
N1—C8	1.439 (2)	C11—C12	1.510 (3)
N1—C10	1.484 (2)	C11—H11A	0.9900
N2—C9	1.331 (3)	C11—H11B	0.9900
N2—C11	1.457 (2)	C12—C13	1.390 (3)
N2—H2N	0.8800	C12—C17	1.394 (3)
C1—C2	1.387 (3)	C13—C14	1.385 (3)
C1—C6	1.400 (3)	C13—H13	0.9500
C2—C3	1.391 (3)	C14—C15	1.382 (4)
C2—H2	0.9500	C14—H14	0.9500
C3—C4	1.385 (3)	C15—C16	1.385 (4)
C3—H3	0.9500	C15—H15	0.9500
C4—C5	1.385 (3)	C16—C17	1.383 (3)
C4—H4	0.9500	C16—H16	0.9500
C5—C6	1.396 (3)	C17—H17	0.9500
			
O2—S1—O1	119.07 (9)	C7—C8—C9	120.57 (17)
O2—S1—N1	108.07 (9)	N1—C8—C9	117.91 (16)
O1—S1—N1	107.75 (9)	O4—C9—N2	122.22 (18)
O2—S1—C1	109.30 (9)	O4—C9—C8	119.70 (18)
O1—S1—C1	109.51 (9)	N2—C9—C8	118.07 (16)
N1—S1—C1	101.71 (9)	N1—C10—H10A	109.5
C7—O3—H3O	109.5	N1—C10—H10B	109.5
C8—N1—C10	115.28 (15)	H10A—C10—H10B	109.5
C8—N1—S1	112.70 (13)	N1—C10—H10C	109.5
C10—N1—S1	116.38 (12)	H10A—C10—H10C	109.5
C9—N2—C11	120.56 (16)	H10B—C10—H10C	109.5
C9—N2—H2N	119.7	N2—C11—C12	112.69 (16)
C11—N2—H2N	119.7	N2—C11—H11A	109.1
C2—C1—C6	122.04 (18)	C12—C11—H11A	109.1
C2—C1—S1	121.72 (15)	N2—C11—H11B	109.1
C6—C1—S1	116.23 (15)	C12—C11—H11B	109.1
C1—C2—C3	118.22 (19)	H11A—C11—H11B	107.8
C1—C2—H2	120.9	C13—C12—C17	118.52 (19)
C3—C2—H2	120.9	C13—C12—C11	122.75 (18)
C4—C3—C2	120.6 (2)	C17—C12—C11	118.69 (18)
C4—C3—H3	119.7	C14—C13—C12	120.5 (2)
C2—C3—H3	119.7	C14—C13—H13	119.7
C3—C4—C5	120.8 (2)	C12—C13—H13	119.7
C3—C4—H4	119.6	C15—C14—C13	120.6 (2)
C5—C4—H4	119.6	C15—C14—H14	119.7
C4—C5—C6	119.8 (2)	C13—C14—H14	119.7
C4—C5—H5	120.1	C14—C15—C16	119.4 (2)
C6—C5—H5	120.1	C14—C15—H15	120.3
C5—C6—C1	118.45 (19)	C16—C15—H15	120.3
C5—C6—C7	121.20 (18)	C17—C16—C15	120.3 (2)
C1—C6—C7	120.22 (17)	C17—C16—H16	119.9
O3—C7—C8	122.42 (18)	C15—C16—H16	119.9
O3—C7—C6	115.26 (17)	C16—C17—C12	120.7 (2)
C8—C7—C6	122.27 (17)	C16—C17—H17	119.6
C7—C8—N1	121.49 (17)	C12—C17—H17	119.6
			
O2—S1—N1—C8	60.59 (14)	C1—C6—C7—C8	−20.3 (3)
O1—S1—N1—C8	−169.54 (12)	O3—C7—C8—N1	−179.44 (17)
C1—S1—N1—C8	−54.42 (14)	C6—C7—C8—N1	3.3 (3)
O2—S1—N1—C10	−163.02 (13)	O3—C7—C8—C9	2.6 (3)
O1—S1—N1—C10	−33.15 (16)	C6—C7—C8—C9	−174.66 (17)
C1—S1—N1—C10	81.97 (15)	C10—N1—C8—C7	−98.7 (2)
O2—S1—C1—C2	106.46 (17)	S1—N1—C8—C7	38.2 (2)
O1—S1—C1—C2	−25.63 (19)	C10—N1—C8—C9	79.3 (2)
N1—S1—C1—C2	−139.44 (17)	S1—N1—C8—C9	−143.81 (14)
O2—S1—C1—C6	−74.54 (17)	C11—N2—C9—O4	−1.5 (3)
O1—S1—C1—C6	153.38 (15)	C11—N2—C9—C8	179.79 (17)
N1—S1—C1—C6	39.56 (17)	C7—C8—C9—O4	6.6 (3)
C6—C1—C2—C3	1.7 (3)	N1—C8—C9—O4	−171.38 (17)
S1—C1—C2—C3	−179.35 (16)	C7—C8—C9—N2	−174.66 (18)
C1—C2—C3—C4	0.1 (3)	N1—C8—C9—N2	7.3 (3)
C2—C3—C4—C5	−0.9 (3)	C9—N2—C11—C12	166.82 (17)
C3—C4—C5—C6	0.0 (3)	N2—C11—C12—C13	−30.1 (3)
C4—C5—C6—C1	1.7 (3)	N2—C11—C12—C17	152.07 (18)
C4—C5—C6—C7	−174.19 (19)	C17—C12—C13—C14	0.2 (3)
C2—C1—C6—C5	−2.6 (3)	C11—C12—C13—C14	−177.66 (19)
S1—C1—C6—C5	178.41 (15)	C12—C13—C14—C15	−0.6 (3)
C2—C1—C6—C7	173.33 (18)	C13—C14—C15—C16	0.7 (4)
S1—C1—C6—C7	−5.7 (2)	C14—C15—C16—C17	−0.3 (4)
C5—C6—C7—O3	−21.9 (3)	C15—C16—C17—C12	−0.1 (3)
C1—C6—C7—O3	162.27 (18)	C13—C12—C17—C16	0.2 (3)
C5—C6—C7—C8	155.6 (2)	C11—C12—C17—C16	178.1 (2)

### Hydrogen-bond geometry (Å, º)

**Table:**

*D*—H···*A*	*D*—H	H···*A*	*D*···*A*	*D*—H···*A*
O3—H3*O*···O4	0.84	1.79	2.531 (2)	146
N2—H2*N*···O1^i^	0.88	2.24	2.980 (2)	141
C10—H10*A*···O4^ii^	0.98	2.50	3.349 (3)	144
C11—H11*B*···O1^iii^	0.99	2.51	3.374 (3)	146
C15—H15···O2^iv^	0.95	2.59	3.496 (3)	158

Symmetry codes: (i) −*x*+1, −*y*+1, −*z*+1; (ii) −*x*+2, −*y*, −*z*+1; (iii) *x*, *y*−1, *z*; (iv) *x*, *y*−1, *z*+1.
